# High Spinal Anesthesia Leading to Cardiac Arrest During Cesarean Section of a Patient With Sickle Cell Disease: A Case Report

**DOI:** 10.7759/cureus.95774

**Published:** 2025-10-30

**Authors:** Ahmad O Hadri, Wouter Ritsema

**Affiliations:** 1 Emergency Medicine, Henry Ford St. John Hospital, Detroit, USA; 2 Anesthesiology, Wayne State University School of Medicine, Detroit, USA

**Keywords:** anesthesia, cardiac arrest, cesarean section, high spine, obstetric anesthesia, spinal anesthesia

## Abstract

High spinal anesthesia is a rare but potentially life-threatening complication during cesarean section. This case report describes a patient who suffered cardiac arrest secondary to high spinal anesthesia. We detail the clinical course, resuscitation efforts, and subsequent management, followed by a discussion of relevant literature and current guidelines. Early recognition and prompt management are crucial for improving outcomes. This report contributes to the body of knowledge on spinal anesthesia complications and emphasizes the importance of multidisciplinary management in such emergencies.

## Introduction

Spinal anesthesia is a commonly used technique for cesarean sections due to its rapid onset, excellent analgesia, and lower maternal and neonatal risks compared to general anesthesia. In the United States, up to 80% of laboring women receive epidural analgesia [1]. While spinal anesthesia offers significant advantages, rare but potentially catastrophic complications can arise. Anesthetic complications are the seventh leading cause of pregnancy-related mortality in the United States, accounting for approximately 1.6% of all pregnancy-related deaths [2].

High spinal anesthesia occurs when the local anesthetic spreads higher than intended, causing severe sympathetic blockade. This can lead to profound hypotension, bradycardia, respiratory depression, and, in extreme cases, cardiovascular collapse.

As one of the most serious anesthetic complications in obstetrics, high spinal anesthesia is a major concern. According to the Society for Obstetric Anesthesia and Perinatology’s SCORE project, the incidence is estimated at 1 in 4,336 neuraxial anesthetics, with cardiac arrest being a rare but significant outcome. Risk factors include obesity, short stature, multiple epidural attempts, and dosing errors [3].

This case report presents a critical instance of high spinal anesthesia leading to cardiac arrest during a cesarean section and discusses prevention and management strategies for anesthesia-related cardiovascular complications.

## Case presentation

Patient demographics

A 28-year-old female, gravida 6, para 5, with a past medical history of sickle cell disease, who was 38 weeks pregnant, presented to the hospital for elective induction of labor. She had two prior uncomplicated vaginal deliveries. On initial presentation, the fetus was cephalic with stable fetal heart tones with moderate variability. A Cook’s catheter was placed at 0900, and the patient was started on low-dose oxytocin for medical induction of labor.

Clinical course

The patient requested epidural analgesia, which was placed by the supervising anesthesiologist at approximately 15:54. The catheter was inserted at the L3-L4 level on the first attempt, with loss of resistance confirmed at 7 cm. A 20-gauge catheter was advanced smoothly and left at 12 cm. Aspiration was performed, with no cerebrospinal fluid (CSF) detected. A test dose of 4 mL of 1.5% lidocaine with epinephrine was administered, followed by an 8 mL bolus of 0.2% ropivacaine. Continuous infusion of ropivacaine was then initiated at 8 mL/hour. Analgesia was titrated to pain using the Visual Analog Scale (VAS), improving from 8/10 to 2/10 within 15 minutes, with stable hemodynamics and no additional boluses required.

The patient was reassessed an hour later, at 16:46, and remained hemodynamically stable, with a blood pressure of 100/54 mmHg, heart rate (HR) of 81 beats per minute, respiratory rate of 18 breaths per minute, and oxygen saturation of 99% on room air. Repeat vitals at 19:45 were similarly stable.

Artificial rupture of membranes was performed at 16:00, revealing a large amount of meconium-stained amniotic fluid. Initially, the patient declined a cesarean section. However, due to recurrent fetal decelerations, the decision was made to proceed with an emergent primary low transverse cesarean section at 21:00.

At approximately 21:10, the ropivacaine infusion was discontinued, and a 20 mL bolus of 2% lidocaine was administered. Upon arrival in the operating room, epidural anesthesia was confirmed as adequate using an Allis clamp to assess sensation. The procedure commenced at 21:23, with the patient’s vital signs remaining stable (blood pressure, 112/55 mmHg; HR, 81 beats per minute; oxygen saturation, 100% on room air).

At 21:25, the patient became dyspneic and was administered 1 mg of lorazepam (Ativan); she was not hypoxic at this time. The fetus and meconium-stained placenta were delivered without complications. However, by 21:26, approximately 15 minutes after the lidocaine bolus, the patient developed increasing weakness, apnea, and bradycardia, followed by asystole.

A Code Blue was called by the CRNA. Chest compressions and bag-mask ventilation were initiated immediately. The patient received 200 µg of phenylephrine and 1 mg of atropine, followed by a 1 mg push of epinephrine. She remained in asystole for approximately two minutes before achieving return of spontaneous circulation at 21:29. She was subsequently intubated for airway protection and started on a phenylephrine infusion.

Differential diagnoses included high spinal block, high epidural spread, and local anesthetic systemic toxicity (LAST). The rapid cephalad progression of weakness, apnea, and bradycardia favored a high neuraxial block rather than LAST, given the absence of neurologic prodrome or arrhythmias.

Outcome

The procedure concluded at 21:49, and the patient was extubated at the end of the case. She was then transferred to the surgical intensive care unit (SICU) for monitoring, where she remained hemodynamically stable on a phenylephrine infusion.

Overnight in the SICU, the patient’s mental status returned to baseline. She remained vitally stable and was successfully weaned off phenylephrine without complications. Electrolytes were monitored and repleted as needed, and she remained on telemetry. A chest X-ray revealed pneumoperitoneum with a small amount of air beneath the right hemidiaphragm, consistent with recent surgery.

The following morning, the patient reported a 20-minute episode of palpitations but denied chest pain, dyspnea, or other concerning symptoms. Cardiology was consulted, and an echocardiogram showed a moderately increased left ventricular cavity size with a normal ejection fraction of 50%. Telemetry monitoring was subsequently discontinued.

The patient tolerated ice chips and clear fluids well and was transferred to the obstetric floor that afternoon for continued postoperative care. She remained hemodynamically stable overnight and throughout the following day. She met all postoperative milestones, ambulated comfortably, and had adequate pain control. After completing a course of antibiotics, she was discharged that evening without any residual concerns.

## Discussion

High spinal anesthesia is a severe complication in obstetric anesthesiology, resulting from unintended cephalad spread of local anesthetic to higher thoracic or cervical levels. This leads to profound sympathectomy, manifesting as bradycardia, hypotension, respiratory paralysis, and, in extreme cases, cardiac arrest due to decreased venous return and cardiac output. This patient’s clinical course illustrates a classic progression of this complication and highlights the importance of vigilant monitoring and prompt intervention. Delayed recognition of symptoms, such as dyspnea, progressive weakness, and hemodynamic instability, can significantly worsen outcomes.

Risk factors

Several factors increase the risk of high spinal anesthesia, including dosing errors, obesity, short stature, repeated epidural attempts, patient positioning (Trendelenburg), and reduced CSF volume in pregnant women [4,5]. Studies have identified obesity as the highest risk factor, followed by multiple failed epidural attempts [2,3]. Additionally, racial disparities have been noted, with the African American race being associated with an increased risk of anesthesia-related maternal mortality [6]. These factors emphasize the need for tailored anesthetic management in high-risk populations.

Early recognition and preventive strategies

Early recognition and proactive management are critical in preventing high spinal anesthesia. Most patients who develop high neuraxial blocks have identifiable risk factors, necessitating a thorough pre-procedural assessment. Standard precautions, including loss of resistance confirmation, aspiration for CSF (despite its limitations), test dosing, and incremental boluses, remain essential safety measures. However, the possibility of needle migration or intrathecal spread, even in the absence of positive CSF aspiration, underscores the need for continued vigilance. A closed claims analysis found that up to 80% of cases involved delays in recognizing or treating high spinal anesthesia, further emphasizing the importance of early symptom identification and preparedness for immediate intervention [7].

Management strategies

Once high spinal anesthesia is recognized, aggressive treatment is required. Management strategies include the administration of vasopressors such as phenylephrine or ephedrine to counteract hypotension, and atropine or epinephrine in cases of severe bradycardia or cardiac arrest [8,9]. Respiratory support, including immediate intubation and mechanical ventilation, is crucial in cases of respiratory compromise. Additionally, patient positioning plays a role in limiting the cephalad spread of anesthetic, with elevating the patient’s head being a useful maneuver [10]. Fluid resuscitation is also vital to maintaining preload and blood pressure.

Consideration in sickle cell disease

In this case, the patient’s sickle cell disease added further complexity to her management. Sickle cell disease predisposes individuals to hypoxia, acidosis, and increased blood viscosity, all of which can exacerbate hemodynamic instability during anesthetic complications. High spinal anesthesia already increases the risk of hypotension and hypoxia, and patients with sickle cell disease may experience even greater adverse effects due to impaired hemoglobin-oxygen affinity and a reduced compensatory reserve. The cardiovascular collapse observed in this case may have been accelerated by the patient’s underlying pathology, as rapid fluctuations in blood pressure and oxygenation are particularly poorly tolerated in this population.

Preventive recommendations

To minimize the risk of high spinal anesthesia, several strategies should be prioritized. Conservative anesthetic dosing is particularly important in high-risk populations, ensuring that patients receive an appropriate and individualized dose. Meticulous monitoring, especially during the initial stages of spinal anesthesia, is crucial to detect early signs of excessive anesthetic spread [11]. Emergency preparedness is essential, with labor and delivery units ensuring ready access to airway management tools and resuscitative medications. Finally, interdisciplinary team training should be emphasized to enhance the early recognition and management of high spinal anesthesia.

## Conclusions

High spinal anesthesia during cesarean delivery is a rare but serious complication that can result in cardiovascular collapse if not promptly recognized and managed. Preventive strategies, including appropriate dosing, patient positioning, and vigilant monitoring, are essential for minimizing risk. In cases where high spinal anesthesia occurs, rapid pharmacologic intervention and respiratory support are critical to preventing fatal outcomes.
